# Supplementary material for: Proton Microbeam Targeted Irradiation of the Gonad Primordium Region Induces Developmental Alterations Associated with Heat Shock Responses and Cuticle Defense in Caenorhabditis elegans
Source: Biology (Basel). 2023 Oct 27;12(11):1372. doi: 10.3390/biology12111372 (PMC10669138; doi:10.3390/biology12111372)
Supplement: Supplementary file 1 [file biology-12-01372-s001.zip › biology-2667552-supplementary.pdf]

Supplementary Data

Proton Microbeam targeted Irradiation of the Gonad Primordium Region Induces Developmental Alterations Associated with Heat Shock Responses and Cuticle Defense in *Caenorhabditis elegans*

Pierre Beaudier [1], Guillaume Devès [1], Laurent Plawinski [1], Denis Dupuy [2], Philippe Barberet [1] and Hervé Seznec [1,*]

1. University Bordeaux, CNRS, LP2I, UMR 5797, 33170 Gradignan, France; beaudier@cenbg.in2p3.fr (Pi.B.) plawinsk@cenbg.in2p3.fr (L.P.); guillaume.deves@cnrs.fr (G.D.); philippe.barberet@u-bordeaux.fr (P.B.); herve.seznec@cnrs.fr (H.S.)

2. University Bordeaux, INSERM, U1212, 33607 Pessac, France; d.dupuy@iecb.u-bordeaux.fr (D.D.)

*Correspondence: herve.seznec@cnrs.fr

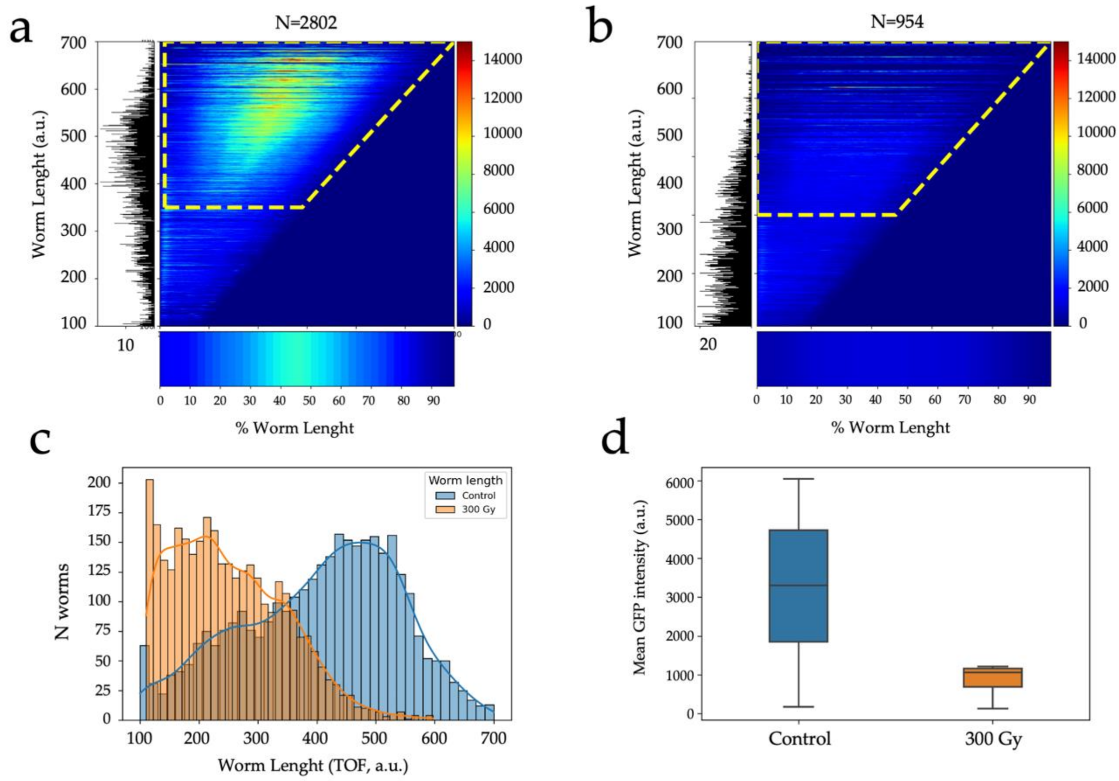

Supplementary Figure S1: Radiation-induced developmental alterations in *C. elegans* following unselective irradiation analyzed by COPAS. (**a**) Longitudinal fluorescence profiles (GFP) in control population as a function of development (worm length, TOF). The appearance of gonads at the L3/L4 stage and above (TOF>250-300, dotted yellow region of interest) is converted into a bar where the calculated mean GFP longitudinal intensity profile is indicated with the length corresponds to the animal's size and the color codes for the fluorescence intensity (bottom figure). The corresponding distribution of worm length is shown on left panel. (**b**) Longitudinal profiles of GFP fluorescence in the population micro-irradiated at 300 Gy. The appearance of gonads at the L3/L4 stage and above (TOF>250-300, dotted yellow region of interest) is visible in calculated mean GFP longitudinal profile (bottom figure). (**c**) Length distribution of worms in control population (blue) and irradiated population (orange). (**d**) Average fluorescence intensity in worms above L3/L4 stage (control, N=*2802;* Gy, N=*954*).

Supplementary Table S1: Radiation-induced differentially expressed genes in *C. elegans* following selective irradiation (300 Gy) analyzed by Long Read Sequencing (Oxford Nanopore technologies).

Gene Symbol	Gene Name	DE Status	Fold Changes	p-value	Identified Pathways
**Heat shock stress**					
**T27E4.8**	hsp-16.1	Up	1.439653399	4.09E-15	Heat shock stress
**Y46H3A.3**	hsp-16.2	Up	1.599351631	7.04E-05	Heat shock stress
**T27E4.3**	hsp-16.48	Up	1.293580584	3.71E-13	Heat shock stress
**T27E4.9**	hsp-16.49	Up	1.567863911	2.41E-10	Heat shock stress
**F44E5.4**		Up	1.231622987	6.94E-06	Heat shock stress, Hsp70 family
**F44E5.5**		Up	1.252968616	5.41E-12	Heat shock stress, Hsp70 family
**F57A8.1**		Up	1.651892213	7.01E-08	Heat shock stress
**F59C12.4**		Up	1.306556184	8.43E-18	Heat shock stress
**R11A5.3**		Up	2.308296289	1.26E-15	Heat shock stress
**Fatty Acid Elongase activity**					
**F11E6.5**	elo-2	Down	-1.114049169	2.19E-06	Fatty acid elongase activity. Involved in determination of adult lifespan; lipid metabolic process; regulation of gene expression.
**F41H10.7**	elo-5	Down	-1.139646916	2.77E-08	Fatty acid elongase activity. Involved in determination of adult lifespan; lipid metabolic process; regulation of gene expression.
**F41H10.8**	elo-6	Down	-1.041064083	3.87E-06	Fatty acid elongase activity. Involved in determination of adult lifespan; lipid metabolic process; regulation of gene expression.
**UDG glucuronosyl transferases family**					
**C10H11.6**	ugt-26	Down	-1.755353953	3.09E-09	Metabolism stress response, UDG glucuronosyl transferases family, involved in chemical detoxification
**Y49C4A.8**	ugt-29	Down	-1.90468465	6.01E-11	Metabolism stress response, UDG glucuronosyl transferases family, involved in chemical detoxification
**Y39G10AR.6**	ugt-31	Down	-1.522264659	6.26E-07	Metabolism stress response, UDG glucuronosyl transferases family, involved in chemical detoxification
**Innate immune activity and signaling pathway**					
**K08D8.5**		Down	-1.257810805	5.11E-07	Innate immune activity and signaling pathway (p38 MAPK)
**F49E11.10**	scl-2	Down	-1.391884051	1.16E-05	Cuticle, Innate immune activity and signaling pathway (p38 MAPK)
**R08B4.5**	nlp-66	Down	-1.231782492	2.47E-06	Cuticle, Innate immune activity and signaling pathway (p38 MAPK)
**Related to gonadal/vulval and epithelial develoment**					
**F23A7.4**		Up	0.704692227	1.59E-07	Vulval lineage trancriptome
**F23A7.8**		Up	1.17361793	1.01E-10	Germ-line specific trancriptome. affected by chemicals exposure
**C52B9.7**	paf-2	Up	1.344196824	4.35E-06	epithelial morphogenesis
**Pathways related to heat and metal induced stress**					
**K11G9.6**	mtl-1	Up	1.816797026	4.51E-08	Heat response, Response to metal induced stress
**C25F9.2**		Up	2.521799072	7.94E-05	Heat stress
**Y38E10A.13**	nspe-1	Down	-1.163088168	2.25E-07	Heat shock stress, arsenic response
**Others pathways**					
**ZK973.6**	anc-1	Up	0.63505832	6.28E-05	Nucleophagy, nucleolar homeostasis (tumor suppressor)
**T22B11.4**	kin-36	Up	1.368273355	1.15E-08	*uncharacterized*
**R08D7.3**	eif-3.D	Down	-0.799341075	1.12E-06	mRNA translation
**C15C8.3**	hrg-7	Down	-1.551418033	1.42E-08	Intestinal heme homeostasis (protease)
**F08F3.7**	cyp-14A5	Down	-1.01822292	5.90E-05	Response to Ionizing Radiation, Phase I detoxification enzyme
**K09E4.4**		Down	-1.282228823	9.55E-06	*uncharacterized*
**C55A1.6**		Down	-0.98767716	8.51E-06	*uncharacterized*
**E04F6.6**		Down	-0.952725622	1.77E-08	*uncharacterized*
**C36H8.3**	flp-9	Down	-0.837940796	9.96E-05	SKN-1 stress pathway
